# Quality of life for children with eosinophilic esophagitis: a comparison of patients’ and parents’ perceptions and associated factors using the PedsQL™ 3.0 Eosinophilic Esophagitis Module

**DOI:** 10.1186/2045-7022-5-S3-P159

**Published:** 2015-03-30

**Authors:** Theadora Swenson, Navneet Virk Hundal, Catherine Chapin, Christine Elliott, John Leung, Aubrey Katz, Paul Hesterberg, Wayne Shreffler, Qian Yuan

**Affiliations:** 1 Massachusetts General Hospital, Boston, MA, USA

## Background

Eosinophilic esophagitis (EoE) is a chronic, immune-mediated disease of eosinophil-predominant inflammation and esophageal dysfunction. The Pediatric Quality of Life Inventory^TM^ 3.0 EoE Module is a validated instrument to measure quality of life (QL) in children ages 2-18 with EoE, but no clinical research studies have been published using this survey. The objectives of this study were to compare QL reported by patients with EoE to that reported by their parents, and to determine which factors predicted QL in this cohort.

## Methods

The module was mailed or emailed to all eligible patients (physician diagnosed EoE while on a PPI medication for 8+ weeks prior to endoscopy and >15 eosinophils/HPF at any level on histopathology) seen in the endoscopy unit from 10/2013-3/2014. QL was reported on a 0-100 point scale with higher scores reflecting better QL. We gathered data on demographics, duration of disease, number of endoscopies, diet therapy, and steroid use. A general linear model approach was used to examine the impact of predictors.

## Results

Thirty-eight percent (23/61) of subjects responded. The average age of the 18 boys and 5 girls was 12 [8-18]. Parent perceived quality of life (PPQL) was 70 [65-78] (median [25%-75%]); child reported quality of life (CQL) was 70 [59-78] and was correlated to PPQL (r=0.697; p<0.001). Disease duration (3y [2-9]) predicted better CQL (3.6 points per year; p=0.003). The significant factors for PPQL were diet and parent's symptoms score of child (PSS). Parents of children on a multi-food elimination diet reported PPQL 12 points lower than the parents of children on milk-only elimination diets (p=0.05). A 1 point improvement in PSS was associated with a 0.5 point increase in PPQL (p=0.006).

## Conclusions

Child and parent QLs were in general agreement; however, there may be important differences between predictors in the two groups. For children, longer duration of disease was associated with better QL and for parents, a more limited diet was significantly associated with poorer QL. Validation of these findings in larger populations is needed, but the results highlight the importance of evaluating QL as a clinical endpoint.

